# Supplementary material for: Metformin Suppresses Stemness of Non-Small-Cell Lung Cancer Induced by Paclitaxel through FOXO3a
Source: Int J Mol Sci. 2023 Nov 22;24(23):16611. doi: 10.3390/ijms242316611 (PMC10705988; doi:10.3390/ijms242316611)
Supplement: Supplementary file 1 [file ijms-24-16611-s001.zip › ijms-2645388-supplementary.pdf]

Supplementary Materials:

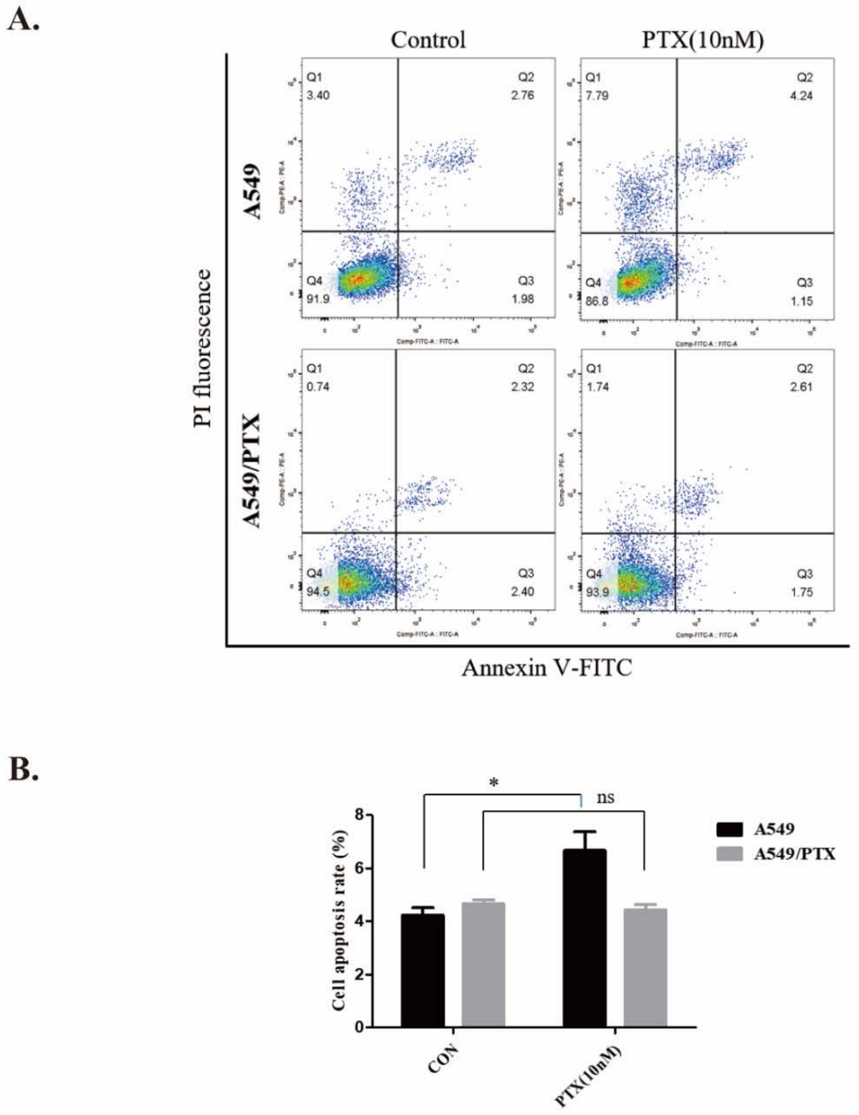

Cell apoptosis rate(%)	Control	PTX
A549	4.20±0.50	6.68±1.22
A549/PTX	4.67±0.25	4.45±0.33

Figure S1. PTX-resistant cells are resistant to paclitaxel-induced cell death. (**A**) A549 and A549/PTX-resistant cells were treated with or without paclitaxel (10 nM) for 48h, and apoptotic cells were detected by Annexin V-FITC and PI staining. (**B**) Cell apoptosis rate was presented according to (**A**). Values are presented as means±SD from three independent experiments, ns means no significant, *p<0.05.

A.

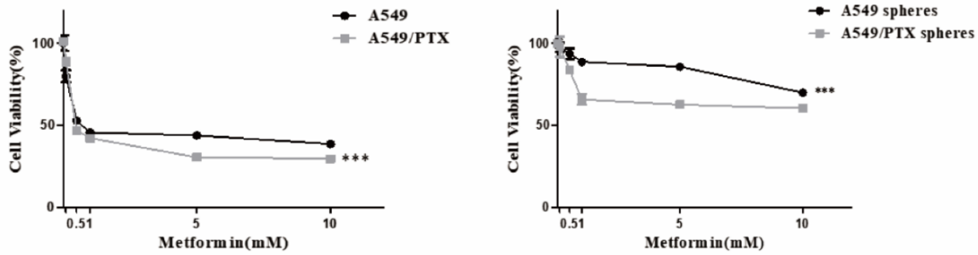

B.

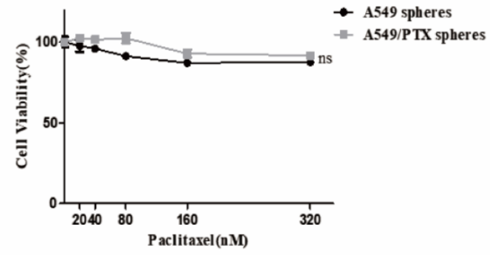

C.

Cell line	IC50 (Paclitaxel)	IC50 (Metformin)
A549	1.19 μM	1.58 mM
A549/PTX	37.56 μM	0.98 mM
A549 spheres	11.00 μM	73.35 mM
A549/PTX spheres	1.44 μM	15.81 mM

D.

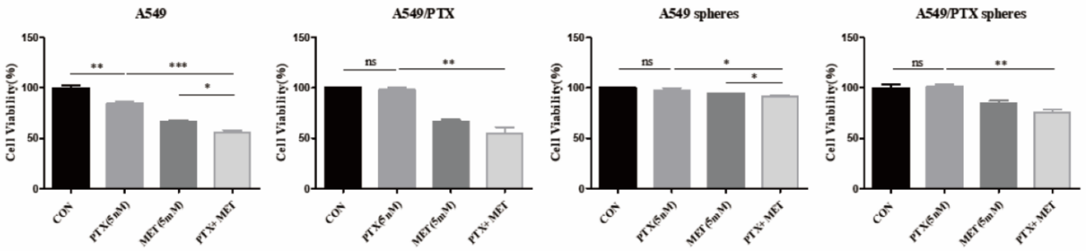

E.

Cell lines	CDI	E_{pre}	E_{exp}	$\Delta E=E_{exp}-E_{pre}$
A549	0.98	0.44	0.45	0.01
A549/PTX	0.84	0.36	0.46	0.10
A549 spheres	0.99	0.07	0.08	0.01
A549/PTX spheres	0.88	0.15	0.24	0.09

Figure S2. PTX-resistant cells are sensitive to metformin but resistant to paclitaxel in 2D or 3D culture. (**A** and **B**) A549 and A549/PTX cells in 2D or 3D culture were treated with metformin (0.1-10 mM) (**A**) or paclitaxel (20-320 nM) (**B**) for 48 h and viabilities calculated as mean ±SD (n=3). (**C**) IC50 values were calculated by the CCK-8 assay. (**D**) A549 and A549/PTX drug-resistant cell lines in 2D or 3D culture were treated with paclitaxel (5 nM), metformin (5 mM) alone or their combination for 48 h and viabilities calculated as mean ±SD (n=3). (**E**) Drug interaction was analyzed using the coefficient of drug interaction (CDI) and Bliss independence (BI) models. CDI<1 or ΔE>0 indicates a synergistic effect, CDI=1 or ΔE=0 indicates an additive effect, CDI>1 or ΔE<0 indicates an antagonistic effect. Data are expressed as the mean ± SD (n=3), ns means no significant, *p<0.05, **p<0.01, ***p<0.001.
